# Seed dormancy release of *Halenia elliptica* in response to stratification temperature, duration and soil moisture content

**DOI:** 10.1186/s12870-020-02560-8

**Published:** 2020-07-28

**Authors:** Da Li Chen, Xin Ping Luo, Zhen Yuan, Meng Jie Bai, Xiao Wen Hu

**Affiliations:** Key Laboratory of Grassland Livestock Industry Innovation, Ministry of Agriculture and Rural Affairs; State Key Laboratory of Grassland Agro-ecosystems; Engineering Research Center of Grassland Industry, Ministry of Education; College of Pastoral Agriculture Science and Technology, Lanzhou University, Lanzhou, 730000 China

**Keywords:** Cold stratification, Temperature, Soil moisture content, Stratification duration, Climate change, Gentianaceae

## Abstract

**Background:**

Although the effect of cold stratification on seed dormancy release has been extensively studied for many species, knowledge of the role of stratifying temperature, soil moisture content and duration of stratification on seed dormancy release at the population level is limited. Here, we aimed to determine the response of seed dormancy release to these factors in six populations of *Halenia ellipti*ca.

**Results:**

Seed dormancy release was more responsive to low than high temperatures, and no dormancy break occurred at 8 °C. Seed germination percentage increased first and then remained unchanged as stratifying soil moisture content increased from 0 to 24%. Seed dormancy release of populations from low altitude was more sensitive to increased stratifying temperature and decreased soil moisture content than those from high altitudes.

**Conclusions:**

Temperature and soil moisture changes resulting from global warming could affect seed dormancy release and consequently seedling establishment. Thus, incorporating data on seed dormancy release involving temperature, soil moisture content and stratification duration is beneficial for predicting plant species regeneration, migration and coexistence in a scenario of climate change.

## Background

Seed dormancy is an adaptive trait and occurs in many angiosperms and gymnosperms [1]. It is defined as the absence of germination of a viable seed in a specified period of time under conditions optimal for germination of non-dormant seeds [2]. Physiological dormancy is common in seeds of summer annuals and many perennials in the temperate zone, and it is can be broken after a period of cold-wet stratification [1, 3].

Temperature during cold-wet stratification, is a critical environmental factor affecting dormancy alleviation [4]. Generally, the effective temperature for seed dormancy release during cold stratification is from ca. 0 to 10 °C depending on the species [1, 5, 6]. For example, dormant seeds of *Carex remota* germinated to a high percentage after 4 weeks stratification at 5 °C, while seed germination was significantly inhibited or even induced secondary dormancy when stratified at 11 °C, 13 °C or 15 °C [7]. In contrast, Kirdar and Ertekin (2008) reported that stratification at 9 °C was more effective than at 4 °C in breaking seed dormancy of four seedlots of *Abies nordmanniana*. The duration of cold stratification also has been found to affect seed dormancy release [1, 8]. The optimal duration of cold stratification to release seed dormancy ranges from 5 days in *Triticum* sp. [9] to 20 weeks in *Stachys alpine* [10]. Seed germination of two species, *Cerastium cerastoides* and *Leucanthemopsis alpina*, were decreased first and then increased as duration of cold stratification increasing [11]. Moreover, a study of grape (*Vitis* spp*.*) seeds showed that dormancy release during cold stratification is controlled not only by the stratifying temperature, but also by the duration of stratification [12], suggesting that the requirement of cold stratification for seed dormancy release is affected by both temperature and its duration.

In addition to temperature and duration of cold stratification, soil moisture content is another key environmental factor affecting seed dormancy release. Previous studies [13–15] have reported that seed dormancy release during cold stratification varies with soil moisture content. Germination percentage of seeds of *Alnus glutinosa* and *Betula pubescens* decreased with increased seed moisture content [14]. On the contrary, seed germination of *Amaranthus retroflexus, Chenopodium album* and *C. hybridum* increased first and then decreased as the soil moisture content was increased during cold stratification [15].

Some studies have determined the response of seed dormancy release to single environmental factors such as temperature [5, 7, 16–18], winter duration [11, 19] and seed/soil moisture content [13–15] during cold stratification. The results of these studies suggested that global warming could impact the dynamics of seed dormancy release and thus delay or enhance plant regeneration from seeds [20–23]. However, climate change is not only a change in temperature, but it also may alter the duration of winter and soil moisture content [20]. Therefore, in a scenario of global climate change, a study of these environmental factors and their interactions during cold stratification on seed dormancy release is meaningful for predicting species distribution, invasion and plant conservation. However, knowledge on the interactive effect of temperature, duration and seed/soil moisture content during cold stratification on seed dormancy release is limited.

Further, temperature, duration and soil moisture content requirements for dormancy release vary among species, and this variation may be an adaptation strategy of species to their specific habitats [15]. Further, species that occur over a wide geographical range may exhibit local adaptations in their specific requirements for dormancy release. Thus, the environmental requirements for cold stratification are expected to differ among populations of a species distributed over a range of local environments.

*Halenia elliptica* D. Don (Gentianaceae) is a subalpine herbaceous biennial, and its freshly matured seeds have physiological dormancy [24]. It is mainly distributed in meadows, forest margins and shrub meadows of the subalpine region (2200–4400 m a.s.l.) on the Qinghai-Tibet Plateau in China [25]. Given that the species occurs over a wide range of elevations, its dormancy-breaking requirements could differ for seeds from different altitudes.

To test our predictions, we collected seeds of *H. elliptica* along an altitude gradient, and addressed the following questions: (i) Do temperature, soil moisture content and their interaction during cold stratification affect seed dormancy release of *H. elliptica* seeds? If so, are there any differences among populations? (ii) Do temperature, duration and their interactions during cold stratification affect seed dormancy release?

## Results

### Effect of temperature and light on germination of fresh seeds

Germination of fresh seeds of all six populations of *H. elliptica* was very low (< 3%) or null at the different temperature tested, both in light and in darkness (Table S1). In addition, a slightly higher germination percentage were observed at 10/20 °C than at 5/15 °C and 15/25 °C for all populations, except for population A (Table S1).

### Effect of temperature and soil moisture content during cold stratification on seed dormancy

**Fig. 1:**
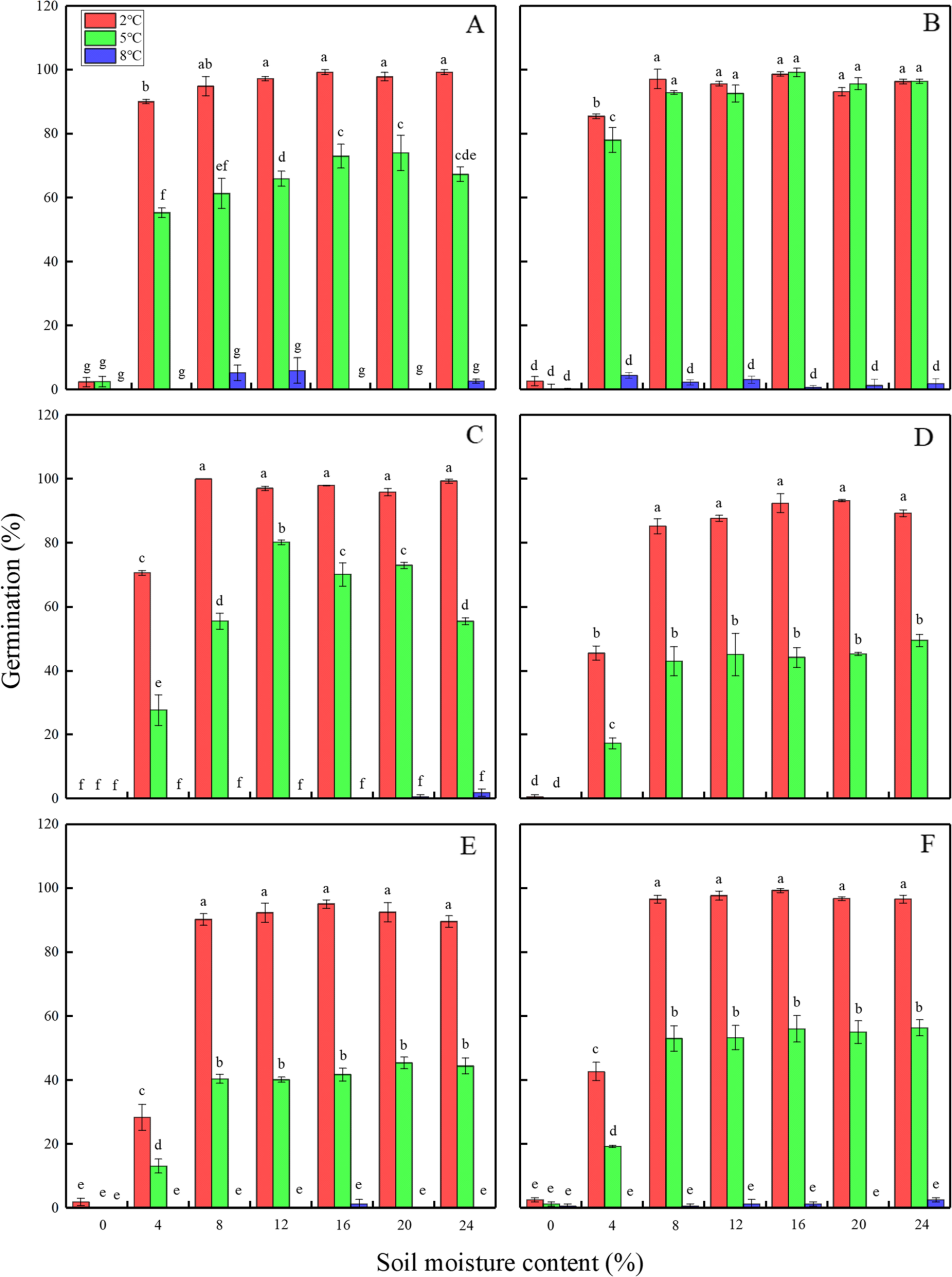
Mean germination percentages for six populations of *H. elliptica* at 10/20 °C (L/D, 12 h/12 h) in response to temperature and soil moisture content during cold stratification (two moths). Different letters indicate significant difference (*P* < 0.05). **a**, **b**, **c**, **d**, **e** and **f** indicate six populations of *H. elliptica* used in this study

Temperature, soil moisture content, population and their interactions, except for temperature × soil moisture content × population, had significant effects on seed germination of *H. elliptica* (*P* <  0.001) (Table 1). Germination percentage of all six populations decreased significantly as stratification temperature increased regardless of soil moisture content (*P* <  0.05). For example, seeds of all six populations germinated to higher than 90% when stratified at 2 °C when soil moisture content was higher than 4%, while they germinated to less than 6% when stratified for 2 months at 8 °C at the same levels of soil moisture content (Fig. 1). Moreover, populations from low altitudes (populations D, E, and F) may be more sensitive to stratifying temperature than those from high altitudes (populations A and C). For example, at 16% soil moisture content, an increase in the stratifying temperature from 2 °C to 5 °C resulted in a decrease in germination from 99 to 73% for population A and from 98 to 70% for population C. In contrast, germination decreased from 93, 95 and 99% to 44, 42 and 56% in population D, E and F, respectively, with a temperature increase from 2 °C to 5 °C at 16% soil moisture content (Fig.1).

**Table 1:** Effect of temperature, soil moisture content, population and their interactions on germination of *H. elliptica* using generalized linear mixed model (GLMM). The asterisk refers to the interaction effect between variables before and after the asterisk

Source	Wald statistic	d.f.	F statistic	F-pr
Temperature (T)	1899.70	2	949.85	< 0.001
Soil moisture content (SMC)	1234.45	6	205.74	< 0.001
Population (P)	835.00	5	167.00	< 0.001
T * SMC	329.25	12	27.44	< 0.001
T * P	134.14	9	14.90	< 0.001
SMC * P	151.75	30	5.06	< 0.001
T * SMC * P	82.93	54	1.54	0.082

Seed germination started when soil moisture content was higher than 4%, as evidence by ≤3% germination of seeds from all stratifying temperatures with 0% soil moisture content. The effect of soil moisture content on seed germination varied with temperature and population. Germination percentage of all six populations, except for population C, increased as soil moisture content increased at 2 °C and 5 °C. There was no significant difference in germination percentage when soil moisture content was higher than 4% (*P* > 0.05). However, seed germination of population C increased and then decreased with increased soil moisture content during 2 months of stratification at 5 °C.

### Effect of temperature and duration during cold stratification on seed dormancy

**Fig. 2:**
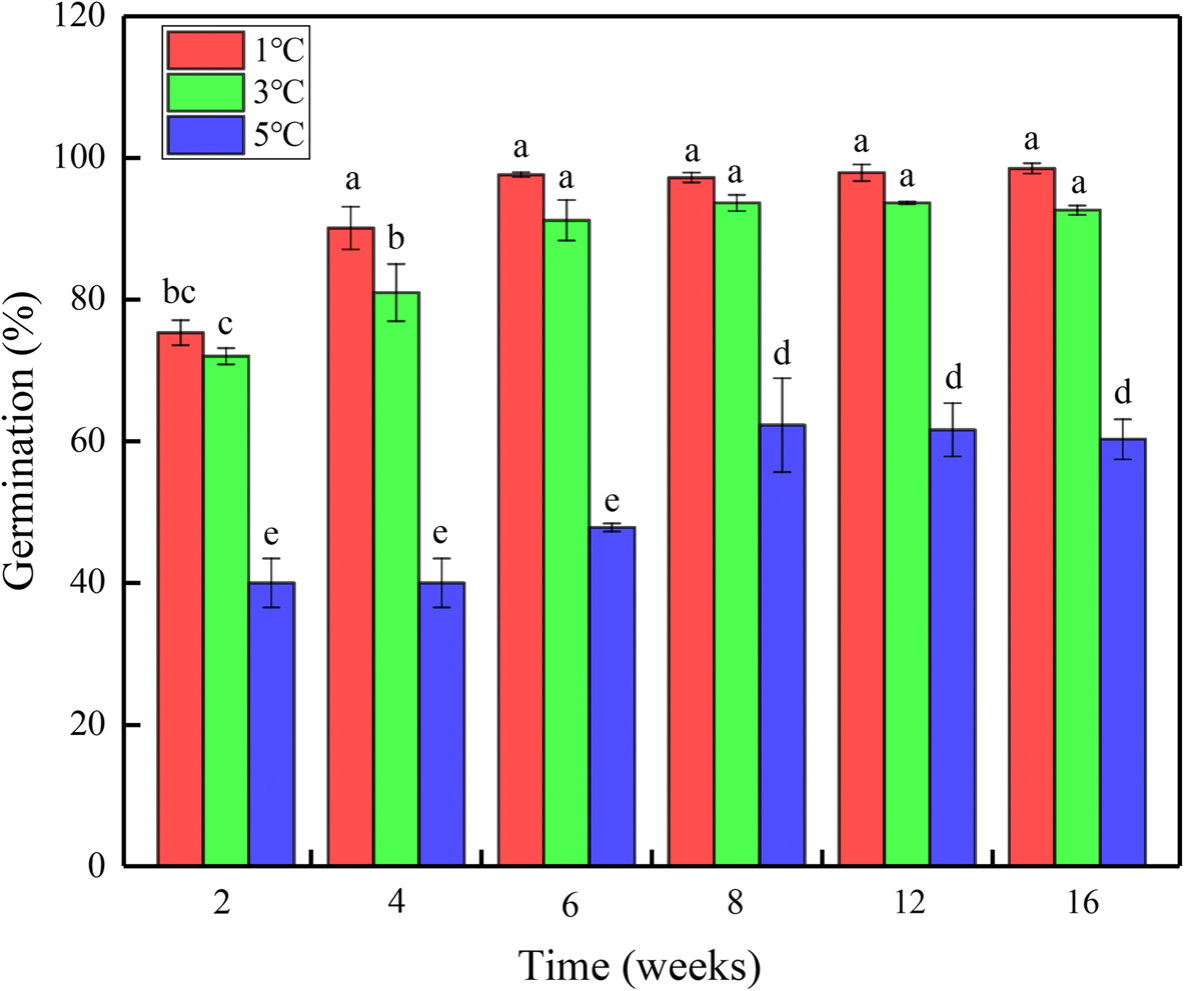
Mean germination percentages for population A of *H. elliptica* at 10/20 °C (L/D, 12 h/12 h) in response to temperature and duration during cold stratification. Different letters indicate significant difference (*P* < 0.05)

Temperature, duration and their interaction had significant effects on seed germination of *H. elliptica* (*P* <  0.001) (Table 2). Germination percentage decreased as temperature increased during cold stratification, regardless of duration. Seed germination increased with increased duration at all temperatures, however, there was no significant difference in germination percentage when the duration was longer than 6 weeks at the same temperature (*P* > 0.05) (Fig. 2). In addition, the cold stratification temperature was set to 1 °C, 3 °C and 5 °C in this experiment, because no or very few seeds germinated after being stratified at 8 °C (Fig. 1). The reason for choosing 16% soil moisture content is that seed dormancy release of population A was not affected by soil moisture content when it was higher than 16% (Fig. 1).

**Table 2:** Effect of temperature, duration and their interaction on germination of *H. elliptica* using generalized linear mixed model (GLMM). The asterisk refers to the interaction effect between variables before and after the asterisk

Source	Wald statistic	d.f.	F statistic	F-pr
Temperature (T)	553.05	2	276.520	< 0.001
Duration (D)	231.15	5	46.230	< 0.001
T * D	60.34	10	6.030	< 0.001

## Discussion

This is the first report on the effect of temperature, soil moisture content and duration of cold stratification on seed dormancy release of different populations of *H. elliptica*. Our study clearly showed that the requirement of temperature and soil moisture content for seed dormancy release differ among populations.

### Effect of temperature and soil moisture content during cold stratification on seed dormancy

Seed dormancy of *H. elliptica* can be released by cold stratification, but the effect varies with the stratifying temperature and soil moisture content. Low temperatures were more effective in releasing dormancy of *H. elliptica* seeds than relatively high temperatures, since germination of *H. elliptica* decreased as stratification temperature increased from 2 °C to 8 °C. This result is consistent with that from a study on *Carex remota* [7] in which seed germination was inhibited as stratification temperature increased from 5 to 15 °C. However, seed germination of *Abies nordmanniana* was significantly increased with an increase of stratification temperature from 4 to 9 °C [16].

The variation in temperature requirements during cold stratification may be attributed to the species difference as well as their habitat [1, 26]. In our research area (> 2200 m a.s.l), seedlings of *H. elliptica* mainly emerged in May (DL Chen, pers. comm.), and the average daily temperature prior to seedling emergence is generally below 5 °C lasting for 5–6 months (Fig. S1). Thus, the temperature in the local area is low enough to release seed dormancy.

Moreover, the requirements of temperature for seed dormancy release of *H. elliptica* differ significantly among populations (*P* <  0.001). Dormancy release of seeds from low altitude populations is more sensitive to temperature increase (from 2 °C to 5 °C) than that of seeds from high altitudes. These results are consistent with those from a study by Kirdar and Ertekin (2008) in which stratification (at 4 °C and 9 °C for 23 days) released seed dormancy of four seedlots of *A. nordmanniana*, however, seeds from the lower altitude (Ortaköy seedlot, 1600 m) showed a greater sensitivity to stratification temperature increase than those from higher altitudes (Meydancık seedlot, 1950 m; Yayla and Veliköy seedlots, 1800 m). Thus, populations from low rather than high altitudes may exhibit inhibition of dormancy release resulting from temperature increases due to global warming.

It is clear from our results that soil moisture content plays a pivotal role in seed dormancy release during cold stratification in seeds of all six populations. Consistent with previous studies [13–15], soil moisture content must reach a critical level for seed dormancy release to occur during cold stratification. In our study, at cold stratification temperatures of 2 °C and 5 °C, seed germination of all six populations begins to occur when soil moisture content was 4% or more. These results suggest that whether seed dormancy can be released mainly depends on temperature and moisture conditions in the field. In our research area, the average soil moisture content from soil surface to subsurface of 7 cm, at altitudes of 2920 m and 3530 m on the eastern of the Qinghai-Tibet Plateau from October to April of the year following seed collection, was 19 and 25%, respectively (Fig. S2). Thus, soil moisture content may not be a limiting factor for dormancy release if seeds are buried below the soil after dispersal. However, seed dormancy may fail to be released if seeds fall on the litter layer or the bare ground where soil moisture content is relatively low [27–29]. Moreover, most seeds of *H. elliptica* were wrapped in sepals and formed an aerial seed bank when they matured, which also may affect seed dormancy release.

It is also worth noting that the requirements of soil moisture content for seed dormancy release of *H. elliptica* differ significantly among populations (*P* <  0.001). The low altitude populations seem to require higher soil moisture content to release seed dormancy than those at high altitudes. For maximum germination percentage, the soil moisture content during cold stratification (at 2 °C) was 8% for population C (high altitude), while it was 20, 16 and 16% for population D, E and F (low altitude), respectively. A possible reason for the difference is that low altitude populations are more likely to suffer drought conditions at the time of spring emergence than high altitude populations, and a high demand for soil moisture content to release seed dormancy may favor seedling survival after emergence at low altitudes. Moreover, a relatively low soil moisture content requirement for dormancy release of high altitudes population will ensure seed dormancy release even in the relative dry winter thus allowing seedlings to emerge early in spring and have all of the warm season for growth [1, 30].

A significant interactive effect between temperature and soil moisture content during cold stratification on seed germination was observed in our study (*P* <  0.001). For example, the requirement of soil moisture content to reach the highest seed germination is higher at 5 °C than 2 °C. A possible explanation is that some biochemical reactions involving seed dormancy release require a certain temperature and soil moisture content, whereas there may be a balance or relationship between these two factors during seed dormancy release [31]. However, the reason for this interactive effect on seed dormancy release requires further study.

### Effect of temperature and duration during cold stratification on seed dormancy

Another consequence of climate change is shortened duration of low temperatures and snow cover, which may affect dormancy break [20, 32, 33]. Seed germination was increased, decreased or unaffected by increased duration of stratification, depending on species [9, 34–37]. In the present study, seed germination increased and then was unot changed as duration of stratification increased at all stratification temperatures, suggesting that a certain minimum time of exposure to low temperature is necessary for dormancy release of *H. elliptica*. Thus, reduction in length of the winter may fail to meet the thermal requirement for seed dormancy release. However, this is not the case for *H. elliptica* since the average daily temperature in our research area from November to April is below 5 °C, and this period is far longer than the duration required for dormancy release. Thus, our results suggest that decreased length of winter may not affect seed dormancy release of *H. elliptica*. This conclusion is consistent with previous research that reduced duration of winter is unlikely to have direct negative impacts on germination or early seedling growth in *Aciphyllya glacialis,* an alpine species from Australia [35].

## Conclusion

Our study clearly shows that temperature, soil moisture content and duration during cold stratification play key roles in regulating seed dormancy release. Further, the requirement of temperature and soil moisture content for seed dormancy release differs among populations. Seed dormancy release of populations from low altitudes may be more sensitive to stratifying temperature increase and soil moisture content decrease than those from high altitudes. These results imply that temperature and soil moisture change resulting from climate change could affect seed dormancy release and consequently seedling emergence and establishment. Overall, incorporating data on seed dormancy release involving temperature, soil moisture content and duration during cold stratification is beneficial for predicting plant species regeneration, migration and coexistence in a scenario of climate change [20].

## Materials and methods

### Seed collection

Freshly matured seeds were collected from six populations of *H. elliptica* growing on the eastern edge of the Qinghai-Tibet Plateau in September 2016 (Plant material used in this study were identified by Kun Liu, who is the associate professor of School of Life Sciences, Lanzhou University, China. A voucher specimen of *H. elliptica* (deposition number PE-02000818) can be examined in the Chinese virtual herbarium at http://www.cvh.ac.cn/spm/PE/02000818. Seeds were collected from the field, and no permission was required to collect them).

Infructescences with ripe seeds were collected from several hundred individual plants at each of the six collection sites (Table 3) and taken to the laboratory, where seeds were separated from other plant material. Seeds were dried at room temperature for 1 week (RH 20–35%, 18–25 °C) and then stored at 4 °C until used in experiments. Thousand seed mass was determined by weighing eight replicates of 100 seeds from each population before experiments commenced.

**Table 3:** Information about the seed collection sites for six populations of *Halenia elliptica* and 1000-seed weight. Different letters indicate significant difference (*P* <  0.05)

Populations	Longitude (E)	Latitude (N)	Altitude (m)	1000-seed weight (g)
A	101.87°	33.68°	3530	1.02 ± 0.03 d
B	102.25°	34.27°	3480	1.16 ± 0.02 c
C	101.99°	33.92°	3470	1.06 ± 0.02 d
D	102.68°	34.91°	3430	1.30 ± 0.02 a
E	102.49°	35.03°	3320	1.06 ± 0.02 d
F	102.51°	35.27°	3310	1.23 ± 0.02 b

### Effect of temperature and light on germination of fresh seeds

To evaluate the effect of temperature and light on germination of mature seeds, freshly collected seeds of all populations (hereafter fresh seeds) were tested at 5/15 °C, 10/20 °C and 15/25 °C in light (12/12 h daily photoperiod, white fluorescent tubes, photon irradiance was 60 μmol m^− 2^ s^− 1^, 400-700 nm) or continuous darkness. For continuous darkness, Petri dishes were covered with two layers of aluminum foil. For each treatment, three replicates of 50 seeds were placed in 10-cm-diameter Petri dishes on two sheets of filter paper (Shuangquan, Hangzhou, China) moistened with 7 ml distilled water. Distilled water was added daily as needed to keep the filter paper moist. Germination of seeds incubated in light were monitored daily for at least 21 days until no further germination occurred for three consecutive days, and a seed was counted as germinated when the radicle was visible (≥ 2 mm). Seeds incubated in the dark were examined for germination only after 28 days. This experiment was performed in October 2016.

### Effect of temperature and soil moisture content during cold stratification on seed dormancy

Seeds of all six populations were mixed with 100 g of soil with a moisture content of 0, 4, 8, 12, 16, 20 and 24% (field capacity = 26%) in a sealed plastic container and cold stratified at 2 °C, 5 °C and 8 °C for 2 months. Because of the limited number of seeds, population D was cold stratified only at 2 °C and 5 °C. For each treatment, three replicates of 50 seeds were used. The determination of field capacity of the soil and different levels of soil moisture content were based on the method of Hu et al. (2018).

Seeds were removed from the soil by using a sieve with 0.5 mm aperture after 2 months of cold stratification and were tested for germination on moist filter paper in light (12 h/12 h) at 10/20 °C, as described above. Seeds were tested for germination only in light (12 h/12 h) for two reasons. Firstly, according to our observation, seeds of *H. elliptica* were wrapped in sepals and formed an aerial seed bank when they matured, most of seeds that fell off the maternal plant remain on the surface even after winter. Secondly, there was no significant difference in germination percentage of cold-stratified seeds under light (12/12 h) and continuous darkness in our preliminary germination test.

### Effect of temperature and duration during cold stratification on seed dormancy

Seeds of one population (population A) were mixed with 100 g of with a moisture content of 16% in a sealed plastic container and keep at 1 °C, 3 °C and 5 °C for 2, 4, 6, 8, 12, 16 weeks. The reason why only population A was used for the experiment was due to the number of seeds of other five populations was limited. After each period of cold stratification, seeds were sieved out from the soil and tested for germination in light at 10/20 °C, as described above. For each treatment, three replicates of 50 seeds were used.

### Data analysis

The effect of incubation temperature, light, population and their interactions and of temperature, soil moisture content, population and their interactions and of temperature, duration of stratification and their interaction on seed germination were tested by fitting generalized linear mixed models (GLMM). Temperature, soil moisture content and population or temperature and duration of stratification were used as fixed effects, while replicates were included as random effects in each model. Seed germination was a probability ranging from 0 to 1, hence we applied a binomial estimation of the model using a logit link function. Tukey’s test was used to compare means when significant differences were found. All data were processed with GenStat, version 18.0 (VSN International Ltd., Hemel Hempstead, UK).

## Supplementary information

**Additional file 1: Table S1.** Effect of incubation temperature and light on germination of freshly-matured seeds.

**Additional file 2: Fig. S1.** Daily mean temperature of MQ and HZ from November to June in 2017–2018 at our research area. Seeds of population A was collected in MQ. The altitude of MQ and HZ were 3530 m and 2920 m in our research area, respectively.

**Additional file 3: Fig. S2.** Soil moisture content of MQ and HZ in our experimental plot. Seeds of population A was collected in MQ. The altitude of MQ and HZ were 3530 m and 2920 m in our research area, respectively. The average soil moisture content from soil surface to subsurface of 7 cm were determined using a TDR 100 (Campbell Scientific Inc., USA) every 2 weeks from October 2017 to April 2018. ** indicate significant difference (*P* <  0.05).

**Additional file 4: **Figure 1. “Germination percentages for six populations of *H. elliptica* at 10/20 °C (L/D, 12h/12h) in response to temperature and soil moisture content during cold stratification (two moths)”. Figure 2**.** “Germination percentages for population A of *H. elliptica* at 10/20 °C (L/D, 12h/12h) in response to temperature and duration during cold stratification”. **Figure S1.** “Daily mean temperature of MQ and HZ from November to June in 2017-2018 at our research area”. **Figure S2.** “Soil moisture content of MQ and HZ in our experimental plot”. Table 1**.** The 1000-seed mass of seeds of the six populations of *H. elliptica*. **Table S1.** Germination percentages of freshly-matured seeds of *H. elliptica*in in response to temperature and light. **Table S2.** The raw datas used in the present study.

## Data Availability

All data generated or analysed during this study are included in this published article (and its supplementary information files, Table. S2).
