# Supplementary material for: Seed dormancy release of Halenia elliptica in response to stratification temperature, duration and soil moisture content
Source: BMC Plant Biol. 2020 Jul 28;20:352. doi: 10.1186/s12870-020-02560-8 (PMC7388213; doi:10.1186/s12870-020-02560-8)
Supplement: Supplementary file 1 — Additional file 1: Table S1. Effect of incubation temperature and light on germination of freshly-matured seeds. [file 12870_2020_2560_MOESM1_ESM.docx]

**Table S1. Effect of incubation temperature and light on germination of freshly-matured seeds.**

Different lowercase letters in the same column indicate significant difference. (*P* < 0.05)

| Populations | Light (h) | 5/15 °C | 10/20 °C | 15/25 °C |
| --- | --- | --- | --- | --- |
| A | 12 | 0.67 ± 0.67 | 0.00 ± 0.00b | 0.00 ± 0.00 |
|  | 0 | 0.00 ± 0.00 | 0.00 ± 0.00 b | 0.00 ± 0.00 |
| B | 12 | 0.00 ± 0.00 | 2.67 ± 1.33 a | 0.67 ± 0.67 |
|  | 0 | 0.00 ± 0.00 | 0.67 ± 0.67 ab | 0.00 ± 0.00 |
| C | 12 | 0.00 ± 0.00 | 0.00 ± 0.00 b | 0.00 ± 0.00 |
|  | 0 | 0.00 ± 0.00 | 0.67 ± 0.67 ab | 0.00 ± 0.00 |
| D | 12 | 0.00 ± 0.00 | 0.00 ± 0.00 b | 0.00 ± 0.00 |
|  | 0 | 0.00 ± 0.00 | 0.00 ± 0.00 b | 0.00 ± 0.00 |
| E | 12 | 0.67 ± 0.67 | 0.67 ± 0.67 ab | 0.00 ± 0.00 |
|  | 0 | 0.67 ± 0.67 | 0.00 ± 0.00 b | 0.00 ± 0.00 |
| F | 12 | 0.00 ± 0.00 | 1.33 ± 0.67 ab | 0.00 ± 0.00 |
|  | 0 | 0.00 ± 0.00 | 0.67 ± 0.67 ab | 0.00 ± 0.00 |
